# Exploring the Role of Serum Hydrogen Sulphide (H2S) Levels in Manic Depressive Psychosis in Terms of Its Association, Diagnostic Ability, and Severity Prediction: Findings From a Tertiary Care Center in North Bengal

**DOI:** 10.7759/cureus.56857

**Published:** 2024-03-24

**Authors:** Sutapa S Dutta, Sayantan Dasgupta, Arup K Banerjee, Indrajit Nath, Utpal Biswas, Nirmal Bera, Alice Ruram

**Affiliations:** 1 Biochemistry, West Bengal Health Service, Kolkata, IND; 2 Biochemistry, North Bengal Medical College and Hospital, Siliguri, IND; 3 Biochemistry, Prafulla Chandra Sen Government Medical College and Hospital, Arambag, IND; 4 Biochemistry, North Eastern Indira Gandhi Regional Institute of Health and Medical Sciences, Shillong, IND; 5 Psychiatry, North Bengal Medical College and Hospital, Siliguri, IND

**Keywords:** gasotransmitter, bipolar disorder (bpd), receiver operating characteristics (roc), hamilton depression rating scale (ham-d), hydrogen sulphide (h2s), manic depressive psychosis (mdp)

## Abstract

Introduction: Manic depressive psychosis (MDP) or bipolar disorder, a prevalent psychiatric condition globally and in the Indian population, has been attributed to various pathological mechanisms. Hydrogen sulphide (H_2_S), a member of the gasotransmitter family, may be linked to the development of bipolar disorder because it plays a crucial role in maintaining proper neuronal function in terms of excitability, plasticity, and homeostatic functions. There is very little data regarding the role of the gasotransmitter H_2_S in MDP in terms of its association, diagnostic ability, and severity prediction, which led us to conduct this study among MDP patients in the Sub-Himalayan region of West Bengal.

Methods: This was an observational case-control study performed in the Department of Biochemistry, North Bengal Medical College and Hospital, Siliguri, West Bengal, India, from January 2022 to December 2022. Fifty diagnosed MDP patients and 50 healthy age- and sex-matched control subjects satisfying the inclusion and exclusion criteria were studied. The H_2_S level in the blood was assayed using the standardised spectrophotometric methylene blue method. The severity of depression was assessed by Hamilton Depression Rating Scale (HAM-D) scoring.

Results: Of the 50 MDP patients, 45 (90%) were in the depressive phase, and five (10%) were in the manic phase. Of the 45 depressive patients, eight (17.8%) had mild depression, 12 (26.7%) had moderate depression, 19 (42.2%) had severe depression, and six (13.3%) had very severe depression. The mean H_2_S level in MDP patients (41.98±18.88 μmol/l) was significantly (P<0.05) lower than that in control subjects (99.20± 15.20 μmol/l). It was also observed that the mean H_2_S level in MDP patients decreased with the duration of the disease but was not statistically significant. The mean H2S levels in the different depression severity groups were found to be significantly different (P<0.001). Receiver operating characteristic (ROC) curve analysis revealed that a cut-off value of H_2_S <78.5 μmol/l was associated with MDP, with a sensitivity of 96% and a specificity of 88%, and a cut-off value of H_2_S < 53 μmol/l predicted the severity of depression with a sensitivity of 89.3% and a specificity of 76.5%.

Conclusion: The significant association of the gasotransmitter H_2_S in MDP patients and its role as a diagnostic and severity predictive marker can help us to employ proper measures for better management of MDP and improving quality of life.

## Introduction

Manic depressive psychosis (MDP), also known as bipolar disorder (BPD), is a very common psychological problem, and 0.5 per 1000 to 21 per 1000 Indians suffer from this disorder [1]. Globally, in 2019, 40 million people experienced BPD [2]. The burden of MDP on the health infrastructure and quality of life of patients is a significant challenge that needs to be addressed holistically. Its impact, mainly on the younger generation, affects the overall productivity of the country. The mean age of onset of bipolar disorder irrespective of its type tends to be between 20-30 years [3].

Although the exact cause of MDP is still unknown, various hypotheses have been proposed about the pathology of this disease. Alterations in the brain structure and signalling systems, along with combinations of genetic and environmental factors, play a significant role in the pathogenesis. To understand the cause of MDP, a new dimension involving the role of gasotransmitters, which play a significant role in neuronal signalling, neuroprotection, and neuromodulation, was evaluated. To date, three gasotransmitters have been detected, i.e., nitric oxide (NO), carbon monoxide (CO), and hydrogen sulphide (H_2_S). H_2_S has emerged as the third endogenous gasotransmitter in the central and peripheral nervous systems. Endogenous H_2_S is produced as a metabolite of homocysteine by cystathionine β synthase (CBS), cystathionine γ-lyase (CSE), and 3-mercaptopyruvate sulphur transferase (3 MST) [4].

H_2_S has recently been identified as a regulator of various physiological events, including vasodilation, angiogenesis, cellular signalling, and antiapoptotic agents [5]. Most patients with MDP are in the depressive phase, which compromises their health and quality of life. Although the neurobiological mechanisms underlying depression have not been fully characterised, emerging evidence suggests a dysfunction in synaptic plasticity in the pathophysiology of depression [6].

Advances in molecular biology research techniques have demonstrated that BPD is associated with alterations in intracellular substances involved in the regulation of neurotransmission, synaptic plasticity, gene expression, and neuronal survival and death [7]. H_2_S, which is an important gasotransmitter, is associated with the maintenance of neuronal plasticity, excitability, and homeostatic functions. Various studies have documented the relationship between reduced H_2_S levels in the hippocampus and the development of MDP [8,9].

H_2_S produced in the central nervous system participates in cognitive function, memory, and neuroprotection. H_2_S has been reported to augment synaptic neurotransmission and facilitate the induction of hippocampal long-term potentiation [10]. Therefore, it is hypothesised from different studies that H_2_S might be a novel antidepressant gaseous mediator [9,11].

Thus, this study aimed to explore the role of serum H_2_S levels in MDP in terms of its association, diagnostic ability, and severity prediction.

## Materials and methods

This was an institution-based observational case-control study conducted at the Department of Psychiatry and Department of Biochemistry, North Bengal Medical College and Hospital, Siliguri, West Bengal, India, from March 2022 to February 2023. A total of 50 patients in the age group of 18-60 years, of either sex, who were clinically stable and properly oriented and who were diagnosed with MDP by the Department of Psychiatry, were included in the study after obtaining proper consent. Another 50 age- and sex-matched, apparently healthy, individuals were included in this study as controls. The study subjects were selected by simple random sampling. Those who had other psychiatric diseases or systemic diseases, such as diabetes mellitus or hypertension, who were taking H_2_S-modifying drugs, e.g., sulphonamides and others, or who were uncooperative were excluded from the study. The study protocol was approved by the Research and Ethical Committee, North Bengal Medical College, Siliguri (approval number: IEC/NBMC/2021-2022/57).

**Figure 1:**
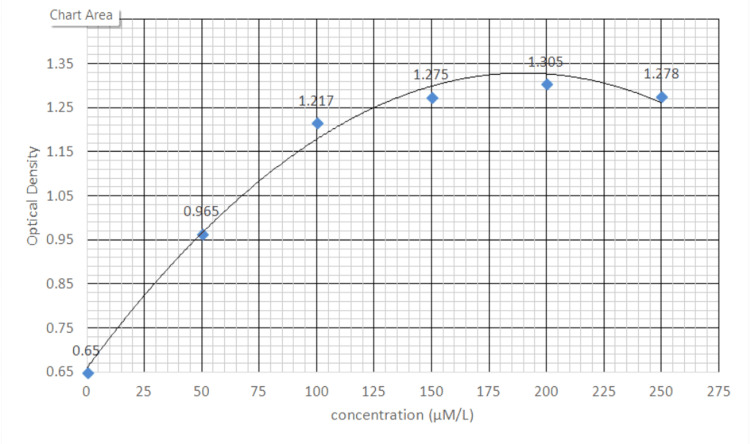
Calibration curve of sodium hydrosulphide (NaHS)

After all aseptic and antiseptic precautions were taken, 5 ml of blood was drawn from the patients’ antecubital veins. H_2_S levels were measured by the modified spectrophotometric (methylene blue) method [12] within one day of sample collection. A quantity of 75 µL of plasma was mixed with 250 µL of 10% trichloroacetic acid and 425 µL of phosphate-buffered saline in a tube. The tube was centrifuged at 3000 rpm for 15 minutes, and 1% (w/v) zinc acetate was added to the supernatant. N-dimethyl-p-phenylenediamine sulphate (20 mmol/L) in 7.2 mmol/L hydrochloric acid (133 µL) and Iron (III) chloride (30 mmol/L) in 1.2 mmol/L hydrochloric acid (133 µL) were also added. Finally, 60 µL of 10% sodium hydroxide was added to the test tube and incubated for 10 minutes at room temperature. The absorbance of the resulting solution at 670 nm was measured with a spectrophotometer. All samples were assayed in duplicate, and the concentration in the solution was calculated against a calibration curve of sodium hydrosulphide (0-250 mmol/L), as shown in Figure 1.

The severity of depression was assessed by the Hamilton Depression Rating Scale (HAM-D), where a score of 0-7 indicated normal, 8-13 indicated mild depression, 14-18 indicated moderate depression, 19-22 indicated severe depression, and > 23 was classified as very severe depression [13]. Two groups were generated based on the severity of depression. MDP with less severe depression comprised those with mild to moderate depression, and MDP with more severe depression comprised those with severe to very severe depression. The other sociodemographic variables were obtained via a proper questionnaire.

Data analysis was performed with the help of IBM SPSS Statistics for Windows, Version 20.0 (Released 2011; IBM Corp., Armonk, New York, United States) and Microsoft Excel 2010 (Microsoft Corporation, Redmond, Washington, United States). The results, which are presented as the means ± standard deviation, were compared between different study groups by applying Student’s t-test for two groups and analysis of variance (ANOVA) for more than two groups. A probability (p) of less than 0.05 was considered significant. A receiver operating characteristic (ROC) curve was generated to determine the cut-off levels of H_2_S.

## Results

The maximum number of patients, i.e., 36% (n=18), was in the age group of 18-28 years, and the minimum number of patients, i.e., 14% (n=7), was in the age group of 51-60 years. Females comprised the highest percentage of cases, i.e., 64% (n=32). Out of the 50 MDP patients, 45 (90%) were in the depressive phase, and five (10%) were in the manic phase. Table 1 shows a comparison of the mean serum H_2_S levels between patients and controls. The decrease in the mean H_2_S level was found to be statistically significant (P<0.001).

**Table 1:** Comparison of the serum hydrogen sulphide level in the study population

Category	No. of cases	Hydrogen sulphide					P-value (Significance 2-tailed)
		Mean	Standard Deviation	Range	Median	Standard Error of Mean	
Cases	50	43.56	±21.80	8–110	35.5	3.08	P<0.001
Control	50	97.52	±17.9	48–156	97.5	2.53	

Table 2 shows the number of cases of MDP grouped according to disease duration and the corresponding mean H_2_S levels; a decreasing trend with increasing duration until the 10th year was noted, but it was determined to be statistically insignificant (P=0.631).

**Table 2:** MDP cases grouped according to disease duration and the corresponding mean hydrogen sulphide levels MDP: manic depressive psychosis

Group according to disease duration (years)	No. of cases (%)	Hydrogen sulphide level (µmol/l) (One-way ANOVA)			
		Mean±Standard Deviation	Degrees of freedom (Df)	Ratio of two variances (F)	P-value
< 1 year	22 (44%)	45±21.09	3	0.58	0.631
1-5 years	21 (42%)	41.48±22.75			
6-10 years	5 (10%)	39±14.35			
>10 Year	2 (4%)	61±43.84			

Table 3 shows the percentage of MDP patients in terms of the severity of depression according to the HAM-D scoring system and the corresponding mean H_2_S level, which showed a decreasing trend with increasing severity, which was found to be statistically significant (P<0.001).

**Table 3:** Percentage of cases in different severity groups and their corresponding mean hydrogen sulphide levels

Depression severity groups	No. of cases (%)	Mean hydrogen sulphide level (µmol/l) (One-way ANOVA)			
		Mean±Standard Deviation	Degrees of freedom (Df)	Ratio of two variances (F)	P-value
Mild depression	8 (17.8%)	74.38±20.02	3	20.08	<0.001
Moderate depression	12 (26.7%)	52.67±11.70			
Severe depression	19 (42.2%)	38.16±14.14			
Very severe depression	6 (13.3%)	21.17±5.56			

**Figure 2:**
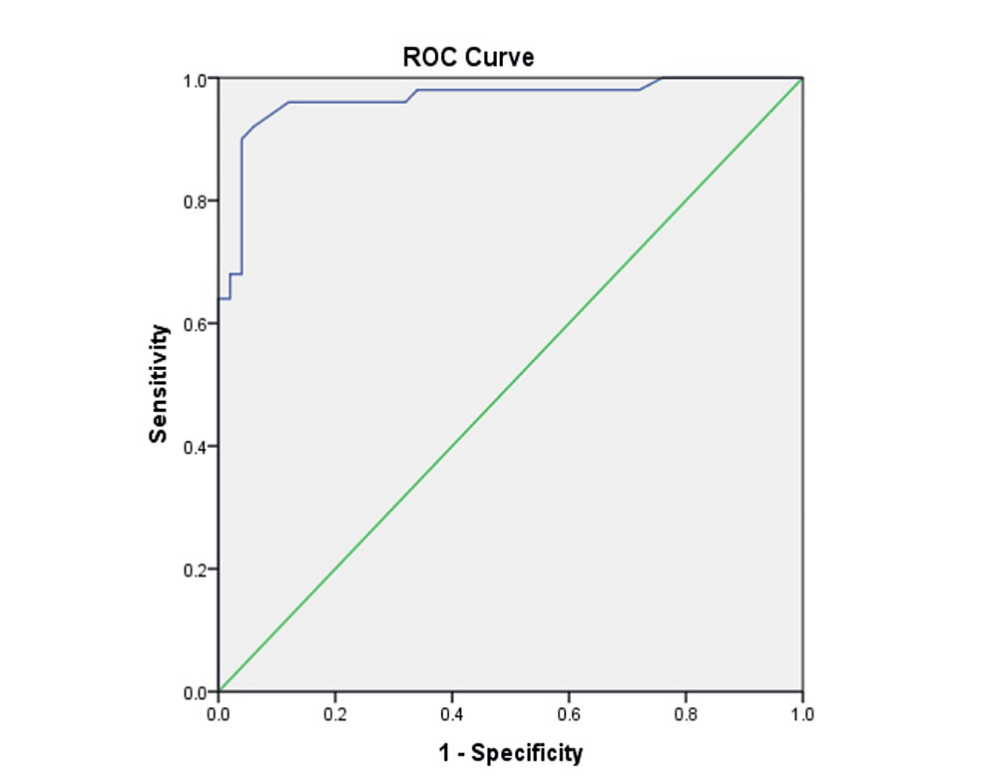
ROC curve of H2S in predicting the occurrence of MDP H2S: hydrogen sulphide; ROC: receiver operating characteristic; MDP: manic depressive psychosis

Figure 2 and Table 4 show the ROC curve of H_2_S in predicting the occurrence of MDP, with an area under the curve (AUC) of 0.964. An H_2_S level of 78.5 µmol/l was taken as the cut-off, below which the presence of MDP can be predicted with a sensitivity of 96% and a specificity of 88%.

**Table 4:** Area under the curve for the ROC curve of hydrogen sulphide amongst cases and controls 78.5 µmol/l is the cut-off value of H2S below which MDP can be predicted with 96% sensitivity and 88% specificity. H2S: hydrogen sulphide; ROC: receiver operating characteristic; MDP: manic depressive psychosis

Test Result Variable(s): H_2_S values				
Area	Standard error	Asymptotic significance	Asymptotic 95% Confidence Interval	
			Lower limit	Upper limit
0.964	.019	.000	.928	1.000

**Figure 3:**
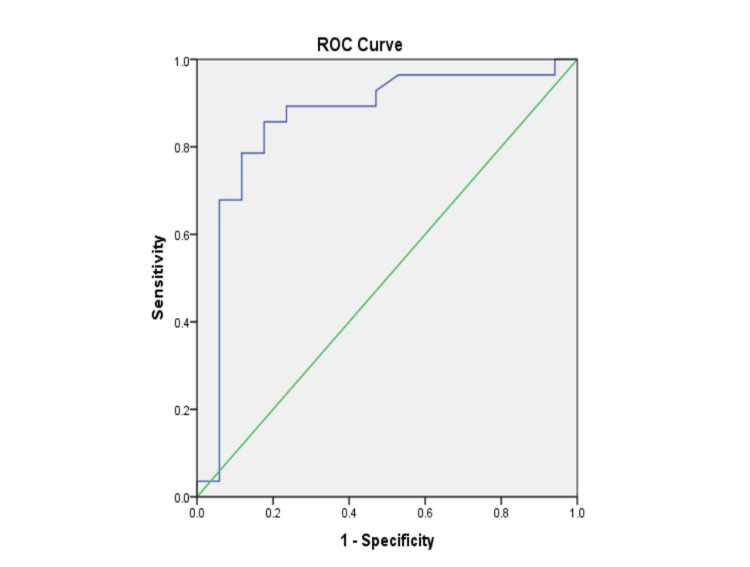
ROC curve of H2S in predicting the severity of depression in MDP. H2S: hydrogen sulphide; ROC: receiver operating characteristic; MDP: manic depressive psychosis

Figure 3 and Table 5 show the ROC curve of H_2_S in predicting the severity of depression in MDP, with an AUC of 0.860. An H_2_S level of 53 µmol/l was taken as the cut-off below which depression in MDP can be considered more severe, differentiating it from less severe depression with 89.3% sensitivity and 76.5% specificity.

**Table 5:** Area under the curve for ROC curve of hydrogen sulphide among different severity groups of depression in MDP 53 µmol/l is the cut-off value of H2S below which MDP can be predicted with 89.3% sensitivity and 76.5% specificity. H2S: hydrogen sulphide; ROC: receiver operating characteristic; MDP: manic depressive psychosis

Test Result Variable(s): H_2_S values				
Area	Standard error	Asymptotic significance	Asymptotic 95% Confidence Interval	
			Lower limit	Upper limit
0.860	0.065	.000	0.733	0.988

## Discussion

In the present study, the greatest percentage of MDP patients (36%) was in the age group of 18-28 years, followed by 32% in the age group of 29-39 years. This finding of MDP being most common in the former age group is in accordance with the study of Bolton et al. conducted in October 2020, which revealed that BPD has a trimodal age-at-onset distribution, divided into the early-, mid-, and late-onset subgroups, with the most common average age at onset being 17.3 years [14].

The above findings can be attributed to the fact that young adults aged between 18 and 28 years experience significant life changes and social strain, which might be the cause of the early onset and incidence of MDP in this age group. In their study, Kozloff et al. reported that genetics, chemical changes in the brain, and environmental factors such as familial stress, substance abuse, and traumatic experiences may contribute to the development of BPD in young adults [15].

The majority of patients in the present study were females (64%). Epidemiologically, we know that MDP is more common in females, an important risk factor for the development of MDP. Other risk factors for MDP include having a family history of the disorder, high stress levels, substance abuse, etc. Meinhard et al. concluded in their article in 2014 that oestrogen fluctuations may be an important factor in the aetiology of BPD [16]. In 2021, Dell'Osso et al. reported a greater preponderance of female patients in every sample and subsample of BPD I and BPD II patients [17]. Compared with males, women with BPD present with greater rates of rapid cycling, depressive polarity, and suicide attempts, which are characteristics of non-inferior severity; prompt recognition and adequate treatment of BPD are therefore crucial for reducing the risk and improving the quality of life of affected women. All the risk factors contributing to the development of BPD were highly prevalent among females in our study population, which mainly comprised the hilly regions of eastern India, leading to the predominant female prevalence of the disease.

In the current study, the mean serum H_2_S level in patients was 43.56 ±21.8 μmol/l, and in controls, it was 97.52± 17.9 μmol/l. A significantly lower H_2_S level was detected in patients (P<0.001). The serum H_2_S level showed a decreasing trend with increasing severity of depression, with a value of 74.38±20.02 μmol/l in mild depression patients and a value of 21.17±5.56 μmol/l in very severe depression patients. This finding is in accordance with a study by Yang et al., which suggested that decreased H_2_S is involved in the pathophysiology of depression and that plasma H_2_S might be a potential indicator of depression severity [18].

Table 2 shows that patients with a duration of illness less than one year comprised the highest percentage (44%), and the lowest percentage (4%) was of those with a duration of illness of greater than 10 years, which indicates that the majority of patients had new-onset disease. The lower number of patients with a duration of illness greater than 10 years can be attributed to the following factors: (i) the patients were taken from the hospital outdoor pool and chosen randomly,(ii) there may be a chance of improvement of symptoms with treatment, and (iii) there may be lower compliance of the patients with seeking medical attention in the long run. When we compared the H_2_S levels in the different study groups according to the duration of disease, a decreasing trend was observed until 10 years, but beyond 10 years, the mean H_2_S value was greater, possibly because of the smaller sample size of that group. These findings suggest that the severity of MDP increases with the duration of the disease. The appropriate H2S level cut-offs in ROC curve analyses can help in the diagnosis of MDP and the prediction of depression severity. It can also aid in the institution of proper treatment strategies for better management of patients.

This study has a few limitations. A single-centre cross-sectional hospital-based study with a smaller sample size limits the applicability of the data obtained from the study for the general population. However, it paves the way for future multicenter, longitudinal studies with larger sample sizes.

## Conclusions

The present study revealed a strong association between H_2_S levels and the occurrence of MDP, which was statistically significant. The serum H_2_S level exemplified the potential to be employed as both a diagnostic tool and a severity prediction tool. Monitoring H_2_S levels and correcting the factors that result in altered H_2_S levels among patients can help in the better management of disease processes and their complications.
